# Analysis of Vicinal Water in Soft Contact Lenses Using a Combination of Infrared Absorption Spectroscopy and Multivariate Curve Resolution

**DOI:** 10.3390/molecules27072130

**Published:** 2022-03-25

**Authors:** Shoichi Maeda, Shunta Chikami, Glenn Villena Latag, Subin Song, Norio Iwakiri, Tomohiro Hayashi

**Affiliations:** 1 Department of Material Science and Engineering, School of Materials and Chemical Technology, Tokyo Institute of Technology, 4259 Nagatsuta-Cho Midori-Ku, Yokohama 226-8502, Japan; maeda.s.am@m.titech.ac.jp (S.M.); chikami.s.aa@m.titech.ac.jp (S.C.); latag.g.aa@m.titech.ac.jp (G.V.L.); song.s.ad@m.titech.ac.jp (S.S.); 2 Life Science Products Division, NOF Corporation, Yebisu Garden Place Tower, 20-3 Ebisu 4-Chome, Shibuya-Ku, Tokyo 150-6019, Japan; norio_iwakiri@nof.co.jp; 3 The Institute for Solid State Physics, The University of Tokyo, 5-1-5, Kashiwanoha, Kashiwa 277-0882, Japan

**Keywords:** infrared absorption spectroscopy, molecular vibration, soft contact lenses, water, multivariate curve resolution, hydrogel, soft material

## Abstract

In this paper, we propose a new spectroscopic method to explore the behavior of molecules near polymeric molecular networks of water-containing soft materials such as hydrogels. We demonstrate the analysis of hydrogen bonding states of water in the vicinity of hydrogels (soft contact lenses). In this method, we apply force to hydrated contact lenses to deform them and to modulate the ratio between the signals from bulk and vicinal regions. We then collect spectra at different forces. Finally, we extracted the spectra of the vicinal region using the multivariate curve resolution-alternating least square (MCR-ALS) method. We report the hydration states depending on the chemical structures of hydrogels constituting the contact lenses.

## 1. Introduction

Water in the vicinity of material surfaces has been studied in many systems since it has significant impacts on various interfacial phenomena such as chemical reaction, adsorption, friction, and adhesion [1,2]. In the field of biomaterials, water at biomaterial surfaces plays a critical role in determining the response to protein molecules, cells, and tissues [3,4,5]. Soft contact lenses are biodevices consisting of polymer hydrogels that contain water molecules inside and also on their surfaces [6,7,8]. Thus, the structure of water in the vicinities of soft contact lenses (SCLs) has been widely studied by a lot of methods, such as sum-frequency generation spectroscopy (SFG) and infrared spectroscopy (IR) [9,10]. However, the OH stretching signal of the vicinal water, which is present at the interface between materials and water, is not accurately and selectively analyzed by conventional methods. For example, sum-frequency generation (SFG) spectroscopy enables us to measure the molecules only in the region where the inversion symmetry is broken. However, it cannot measure the whole interfacial region with several layers of interfacial molecules. As for attenuated total reflection (ATR) infrared (IR) absorption spectroscopy, it measures both interface and bulk regions due to the long evanescent field’s decay length (several hundred nm) [11,12]. Therefore, a method is required to analyze structures of vicinal water, whose property is different from bulk [13,14].

Here, we apply the algorithm of multivariate curve resolution-alternating least squares (MCR-ALS) to resolve overlapping OH stretching signals of water molecules into two components, namely bulk and vicinal water. MCR-ALS is a model-free or soft-modeling method that extracts pure component contributions from multicomponent components [15]. All that is needed to perform an analysis with MCR-ALS is matrix data of raw measurement results that contain a mixture of the components. MCR-ALS can provide information on the concentration and spectral profile of each component. Thus far, MCR-ALS has been successfully used to analyze data obtained from a variety of analytical techniques, including mass spectrometry [16,17,18], spectroscopic techniques [10,19,20], and cyclic voltammetry [21,22,23].

In this study, a straightforward method for measuring the vibrational spectra of the vicinal water of water-containing soft materials, such as SCLs, has been developed by combining attenuated total reflection-infrared spectroscopy (ATR-IR) with controlled pressure and the MCR-ALS method. Multiple ATR-IR spectra with varying signal ratios of bulk and vicinal water were obtained through dehydrating the SCLs by changing the pressure applied to the SCLs. Using the MCR-ALS method to the ATR-IR spectra of water in the vicinity of SCLs, the OH stretching signals were resolved into two components (i.e., bulk and vicinal water). We attempted the comparison of the spectra of the vicinal water of four different SCLs. This comparison shows that the difference in spectra depends on the composition of the materials that compose the SCLs. Moreover, it was found that there is a material dependence of the spectra of vicinal water. In this paper, we also discussed the relationship between the degree of fouling and the spectral shapes of the vicinal water in poly(hydroxyethyl methacrylate) (PHEMA)-based SCLs and silicone-based SCLs.

## 2. Materials and Methods

### 2.1. Soft Contact Lenses (SCLs)

The SCLs analyzed in this work are summarized in Table 1. The SCLs (PHEMA-based) of nonionic and anionic hydrogel used for were omafilcon A (Group II) and ocufilcon D (Group IV), commercially available as Proclear^®^ 1 day (CooperVision Inc., San Ramon, CA, USA) and Menicon^®^ 1 DAY (Menicon Co., Ltd., Nagoya, Japan), respectively. In addition, the SCLs (silicone-based) of nonionic hydrogel (Group V) used were delefilcon A and somofilcon A, commercially available as DAILIES TOTAL 1^®^ (Alcon Inc., Geneva, Switzerland) and clariti^®^ 1 day (CooperVision Inc., San Ramon, CA, USA), respectively.

### 2.2. Attenuated Total Reflection—Infrared Spectroscopy (ATR-IR)

The IR absorption spectra of hydrated SCLs were measured by Fourier-transform infrared (FTIR) absorption spectrometer (FT/IR-4600, JASCO Inc., Tokyo, Japan) equipped with a diamond prism for an ATR configuration. The measurements were performed at room temperature, and the measurement chamber was constantly purged by pure nitrogen gas. The intensity of the evanescent field generated in the vicinity of the prism surface decreases exponentially away from the surface. The decay length of the evanescent wave, *d* is expressed as Equation (1):

(1) $$d=\frac{\lambda }{2\pi n_{1}\;\sqrt{\sin^{2}\theta -{\left(\frac{n_{2}}{n_{1}}\right)}^{2}}}$$

where *n*_1_, *n*_2_, *λ*, and *θ* are the refractive indices of the diamond prism and sample, the wavelength of the infrared light, and the incident angle, respectively. All spectra were collected in the regions from 4000 to 500 cm^−1^ with a resolution of 4 cm^−1^, and 300 spectra were averaged to acquire a final spectrum. In this work, we focus on the region between 3800 and 2800 cm^−1^ (region of OH stretching mode), where the theoretical penetration depth ranges between 416.3 and 570.0 nm.

**Figure 1:**
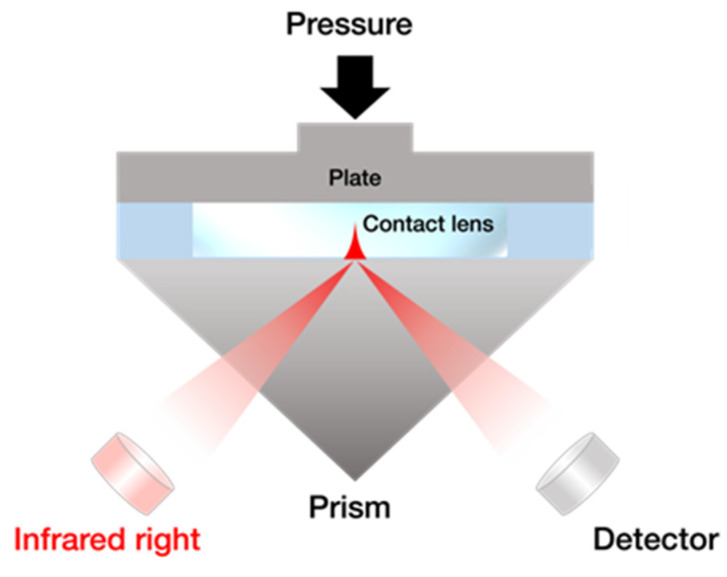
Schematic illustration of the pressure-controlled ATR-IR method.

**Figure 2:**
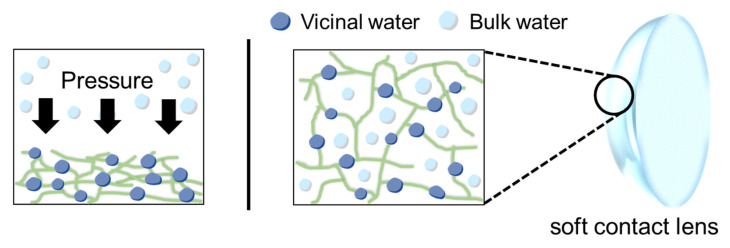
The dehydration process of SCLs by applying pressure.

The background spectrum on the prism without the solution was taken first, and then 10 μL of the filling solution was put onto the prism to obtain the spectrum of the bulk water. After that, a piece of the SCLs cut into 5 × 5 mm^2^ surrounded by a plastic wall was placed in the solution on the prism, then the pressure-controlled ATR-IR measurements were performed by pressing down the SCLs with a plate from the top, shown in Figure 1. In this configuration (Figure 1), we can minimize the drying of the SCLs during the measurements. In that case, the ATR-IR spectra include three different signal components, i.e., (i) bulk, (ii) water near the polymer networks of the SCLs (vicinal water), and (iii) interfacial water between the prism and SCLs. The contribution of (i) decreases, whereas the contribution of (ii) increases when the pressure to hold down the SCLs is increased due to dehydration of SCLs (Figure 2). The ATR-IR spectra, which consist of bulk and the vicinal water between the SCLs and the prism, were collected t at different pressures [Pressure 1 to Pressure 4 (ranging between 26 and 157 kPa), with the latter having the highest pressure exerted]. We optimized the applying pressure to avoid irreversible damage to the SCLs through this process. We checked that there is no damage to the SCLs from the recovery of the shape of the SCLs by optical microscopy and the reproducibility of the ATR-IR spectra.

### 2.3. Multivariate Curve Resolution—Alternating Least Squares (MCR-ALS)

**Figure 3:**
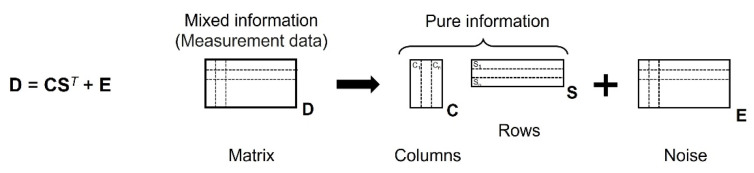
A principle of multivariate curve resolution (MCR).

The MCR-ALS process was performed in two steps with MATLAB 2021b using the MCR-ALS package developed by Jaumot et al. [24,25]. The data of all ATR-IR spectra in the region between 3800 and 2800 cm^−1^ (OH stretching band) were imported into MATLAB. The initial estimation was calculated by a principal component analysis to separate the spectrum into two pure components at the first step of the MCR-ALS method with the non-negativity restrictions. At the second step of the MCR-ALS process, the difference spectra were calculated to remove the components of (iii). The data from the calculation of the difference spectra can be arranged in a data matrix **D** (*r* × *c*), the *r* rows of which are the number of the difference spectra and the c columns of which are the number of absorbance wavelengths. The MCR-ALS decomposition of matrix **D** is carried out according to Equation (2):

**D** = **CS***^T^* + **E** (2)

where **C** (*r* × *n*), **S*^T^*** (*n* × *r*), and **E** (*r* × *c*) are the matrix that describes how the *n* chemical species’ contribution in the spectroscopically active process varies in the different *r* rows of the data matrix, the matrix that describes how the spectra of *n* species changes in the c columns of the data matrix (pure spectra profiles) estimated by the initial estimation in the first step of the MCR-ALS method, and the residuals matrix with the data variance that cannot be explained by the product **CS*^T^***, respectively. This relationship is shown in Figure 3. The bulk and vicinal water spectra were estimated from the initial estimation analysis in this study. Then, we apply the MCR-ALS method to extract spectra of two components from the difference spectra of mixtures of bulk water and vicinal water.

## 3. Results and Discussion

**Figure 4:**
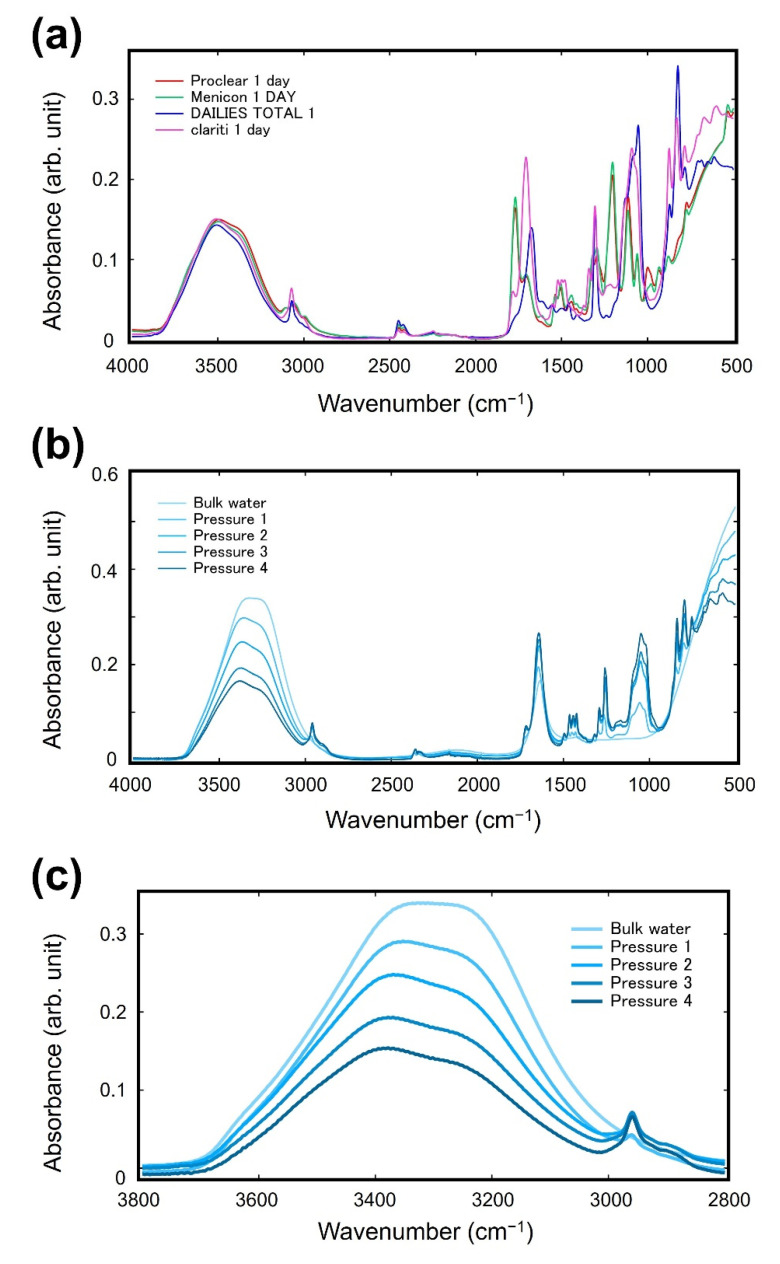
(**a**) ATR-IR spectra of the SCLs in the whole measuring range with applying pressure 4; (**b**) ATR-IR spectra in the whole measurement region under different pressures. (clariti^®^ 1 day) (**c**) ATR-IR spectra in the region of the OH stretching band under different pressures (clariti^®^ 1 day).

Figure 4a displays typical ATR-IR spectra of contact lenses in the whole measurement range with applying pressure 4 (157 kPa). Depending on the monomers constituting the SCLs, the spectral shapes in the fingerprint region (1500–500 cm^−1^) are different. Figure 4b shows the spectra measured under different pressure (clariti^®^ 1 day). With increasing the pressure (Pressure 1 to Pressure 4), the signal in the fingerprint region increases, whereas the intensity in the O-H stretching region decreases. This indicates that the application of pressure induces the dehydration of SCLs in the measuring region. Figure 4c shows the spectra in the OH stretching region under different pressure (clariti^®^ 1 day). Together with the decrease in the intensity, the spectral shape also changed, indicating that the ratio between the vicinal and bulk water changes depending on the applying pressure. As the applied pressure increased, the dehydration of SCLs progressed.

**Figure 5:**
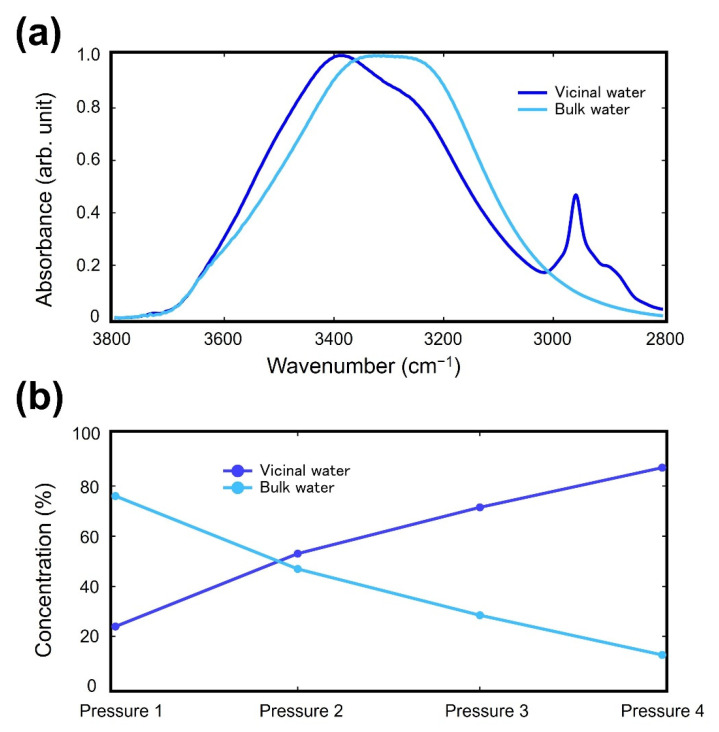
The results of (**a**) ATR-IR spectra and (**b**) concentration ratio by MCR-ALS analysis (clariti^®^ 1 day).

We applied the MCR-ALS method to the spectra in the OH stretching region to separate the spectral components of bulk and vicinal water. Figure 5a displays the results of ATR-IR spectra, and Figure 5b displays the concentration of bulk and vicinal water obtained by the MCR-ALS method. We successfully resolved the overlapping ATR-IR spectra of bulk and vicinal water. The peaks in the region between 2900 and 3000 cm^−^^1^ are assigned to CH stretching modes. Since these modes are not included in the spectrum of bulk water, these modes are extracted together with the components of the vicinal water in the MCR-ALS process.

The MCR-ALS process also provides the ratio of the components (in this case bulk and vicinal water). Figure 5b showed that the ratio of the vicinal water increases, whereas that of bulk water decreased with the increase of the pressure, which is reasonable considering the process of dehydration under the application of pressure.

**Figure 6:**
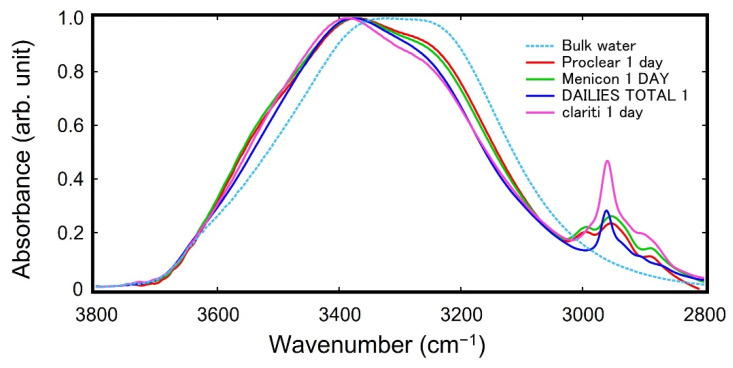
ATR-IR spectra of vicinal water of SCLs in the OH stretching region obtained by the MCR-ALS method. Proclear 1 day and Menicon 1 DAY are PHEMA-based SCLs, and DAILIES TOTAL 1 and clariti 1 day are silicone-based SCLs.

Figure 6 shows the spectra of bulk water and vicinal water of each sample in the OH stretching region. The spectra in the OH stretching region reflect the state of hydrogen bonding states of the vicinal water determined by water–water and water–SCLs interactions. The spectra of the vicinal water of PHEMA- and silicone-based SCLs were different from that of bulk water. This indicates that the hydrogen bonding state of the vicinal water is different from that of bulk water due to the water-SCLs interaction.

**Figure 7:**
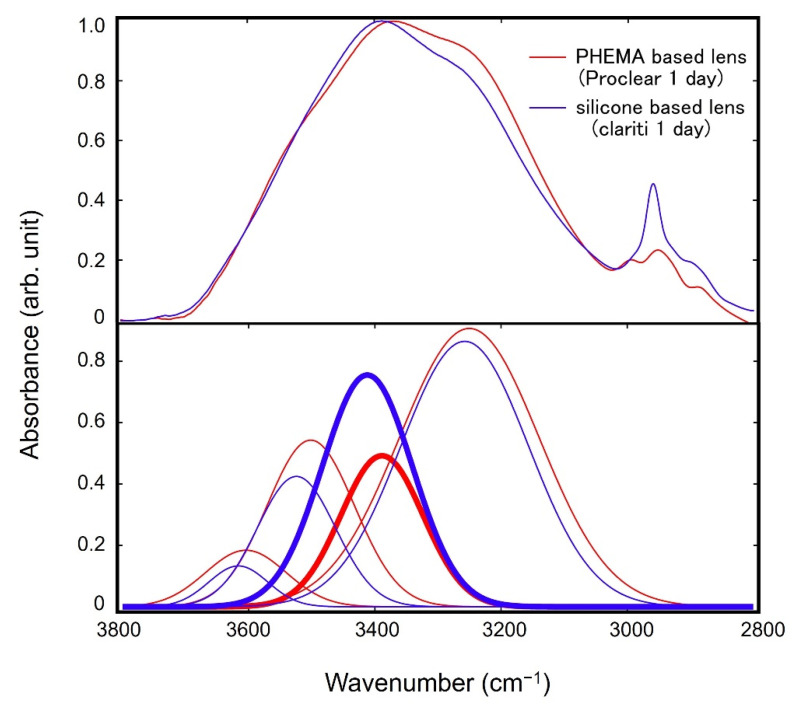
The results of the peak fitting of the spectra of the vicinal water for PHEMA (Proclear 1 day) and silicone-based (clarity^®^ 1 day) SCLs. The peaks for the CH stretching modes in the region between 3000 and 2900 cm^−^^1^ were removed by assuming peaks for the modes in the fittings.

We performed the peak fitting to deconvolute the spectra into pure components to extract more information from these spectra. Figure 7 shows the results of the peak fitting of the spectra of the vicinal water for PHEMA and silicone-based SCLs. For the fitting, we checked the second derivative of each spectrum to find the position of the peaks in the O-H stretching region. For all spectra, we found four major components at around 3200, 3400, 3500, and 3600 cm^−^^1^, and we performed the fitting of the spectra with the four peaks by optimizing the positions of the peaks.

According to the previous works on the analyses of water in the vicinity of polymeric materials by IR absorption spectroscopy [12], water can be classified into three categories: water that is tightly hydrogen-bonded to polymers, water which is weakly bound to polymers, and water which has no hydrogen bond with polymers and has a bulk water-like structure. These three types in the classification are often denoted as non-freezing (strongly bound) (peak at around 3600 cm^−^^1^), freezing bound (weakly bound) (3400 cm^−^^1^), and freezing (free) (3200 cm^−^^1^) water, respectively.

The most prominent difference is found for the intensity of freezing bound water at around 3400 cm^−^^1^. Freezing bound water has flexible mobility and fewer water clusters than bulk water, playing an essential role in anti-fouling properties by acting as a water barrier when proteins and cells adsorb onto polymers [26,27,28]. Freezing bound water has been observed in blood compatible polymers such as poly(2-methoxyethyl acrylate) (PMEA), poly(ethyleneeglycol) (PEG), and poly(2-methacryloyloxyethyl phosphorylcholine) (PMPC) [12]. In general, it is well-known that the amounts of protein adsorbed on SCLs are less for silicon-based SCLs than PHEMA-based ones [8]. Our results show that the peak tops of the vicinal water spectra of silicone-based SCLs with higher protein resistance are at around 3400 cm^−^^1^, indicating agreement with the above-mentioned previous findings on blood-compatible polymeric materials.

We are currently measuring the amount of protein adsorption on SCLs quantitatively and correlating the spectra of the vicinal water with the degree of the SCLs’ protein resistance. Furthermore, we will also attempt to connect the water structure with the degree of amenity during the usage of SCLs since the vicinal water contributes to friction between an eye surface and SCLs.

## 4. Conclusions

In this work, we proposed a method to analyze the behavior of water in the vicinity of water-absorbing soft materials such as hydrogels by using conventional ATR-IR absorption spectroscopy by applying various pressures to modulate the ratio between interface and bulk components. Then, we extract these spectral components using the MCR-ALS method from the set of measured spectra. Our results showed that the spectral shapes of the OH-stretching band, which reflects the hydrogen bonding state of the vicinal water, are different depending on the chemical structure of polymers constituting contact lenses. We found that our method can effectively explore the hydration states of materials functioning in water, such as porous materials, hollow fibers, and other soft materials.

The modulation to the ratio of vicinal and bulk components is not limited to the pressure used in this work. When we employ the ATR-IR configuration, the concentration of samples or the surface-prism distance can be possible modulation to change the ratio between bulk and interface components. We are currently evaluating various interfaces using these modulations, which will be published elsewhere.

## Figures and Tables

**Table 1:** SCLs used in the experiments.

Trade Name	USAN Name	FDA Category (Group)	Constituent Monomers
Proclear^®^ 1 day	omafilcon A	II	HEMA, MPC
Menicon^®^ 1 DAY	ocufilcon D	IV	HEMA, MAA
DAILIES TOTAL 1^®^	delefilcon A	V	silane, NNDMAA
clariti^®^ 1 day	somofilcon A	V	silane, NVP

USAN = U.S. Adopted Names; FDA = Food and Drug Administration; Group Ⅱ = high water (≥50%), nonionic; Group Ⅳ = high water (≥50%), ionic; Group V = silicone-based; HEMA = hydroxyethyl methacrylate, MPC = 2-Methacryloyloxyethyl phosphorylcholine, MAA = methacrylic acid, NNDMAA = N,N-dimethylacrylamide; NVP = N-vinylpyrrolidone.

## Data Availability

Our research activities are summarized in http://lab.spm.jp/.
